# Functional and radiological outcomes of a minimally invasive surgical approach to monostotic fibrous dysplasia

**DOI:** 10.1186/s12957-016-1068-1

**Published:** 2017-01-05

**Authors:** Mamer S. Rosario, Katsuhiro Hayashi, Norio Yamamoto, Akihiko Takeuchi, Shinji Miwa, Yuta Taniguchi, Hiroyuki Tsuchiya

**Affiliations:** 1 Department of Orthopaedic Surgery, Kanazawa University School of Medicine, 13-1 Takara-machi, Kanazawa, Ishikawa 920-8640 Japan; 2 Department of Orthopaedics, East Avenue Medical Center, East Avenue, Diliman, Quezon City, 1101 Metro Manila Philippines

**Keywords:** Fibrous dysplasia, Prophylactic bridge plating, Minimally invasive approach, Alpha-tricalcium phosphate, Open biopsy

## Abstract

**Background:**

Reports showing high recurrence rates for intralesional curettage and bone grafting have made the current treatment principle for fibrous dysplasia controversial. This study aimed to report the postoperative clinical outcomes from three minimally invasive surgical strategies we use for monostotic fibrous dysplasia (MFD).

**Patients and methods:**

Twelve patients with MFD presenting with no pathologic fracture or deformity and treated with one of three surgical strategies—plain open biopsy, plain alpha-tricalcium phosphate (ATP) reconstruction, and prophylactic bridge plating—were included. There were nine men and three women, with median age of 38 years. Mean follow-up was 88 weeks. Five cases involved the proximal femur, two each involved the femoral and tibial diaphyses, and one each involved the distal humerus, radial diaphysis, and proximal tibia. All cases were reviewed for functional and radiological outcomes.

**Results:**

Median time to full activity was 1 day (range 1 to 3) for the plain open biopsy group, while the prophylactic bridge-plating and plain ATP reconstruction groups had longer median recovery times (59 days, range 3 to 143, and 52 days, range 11 to 192, respectively). Musculoskeletal Tumor Society scores at last follow-up were excellent for all the cases (mean 29.6, range 25 to 30). Radiological analysis using Gaski et al.’s criteria showed plain open biopsy resulted in partial resolution of proximal femoral lesions, while ATP reconstruction and prophylactic plating resulted in no change and progression in this lesion site, respectively. For femoral diaphyseal lesions, prophylactic plating resulted in partial resolution, while ATP reconstruction resulted in no change. In the tibial diaphysis, prophylactic plating resulted in partial resolution, while plain open biopsy resulted in no change. For the lesions involving the distal humerus and the proximal tibia, plain open biopsy resulted in partial resolution, while for the radial diaphyseal lesion, ATP reconstruction resulted in no change. Radiological progression was limited in 11 (92%) cases, and none had postoperative complications.

**Conclusion:**

Plain open biopsies for asymptomatic lesions; prophylactic bridge plating for symptomatic, large diaphyseal lytic lesions; and plain ATP reconstructions for both small and large nondiaphyseal symptomatic lytic lesions may be acceptable alternatives to curettage-incorporating procedures for MFD.

## Background

Fibrous dysplasia (FD) is a developmental, nonneoplastic disorder that is associated with mosaic somatic activating mutations in *GNAS*, which encodes the cAMP pathway-associated G-protein, G_s_α, and affects tissues derived from the ectoderm, mesoderm, and endoderm [1]. In skeletal tissues, bone maturation arrest at the woven bone stage that is caused by the disease is characterized by widening of the affected bone with cortical thinning and the presence of fibro-osseous tissue [2]. There is a wide range of involvement and it can vary from an isolated single lesion in a single bone (monostotic) to involvement of many bones (polyostotic), if not the entire axial and appendicular skeleton [3]. The polyostotic form may also be part of a separate assemblage of symptoms describing both McCune-Albright [4] and Mazabraud syndromes [5]. Symptoms usually appear between 5 and 20 years of age, with earlier onset seen in extended cases of dysplasia [5]. Increased G_s_α signaling is the underlying cause of the bone and skin lesions, accelerating both the osteogenic commitment of bone marrow stromal cells but inhibiting their further differentiation into osteoblasts [6] and the action of alpha-melanocyte-stimulating hormone (alpha-MSH) to stimulate melanin production [7], respectively.

Limp, pain, and pathologic fracture are the usual presentations of FD [3]. Lesions can expand and deform bone, thereby threatening structural stability [3]. In a study of 172 fractures in patients with FD, peak fracture rate was observed between ages 5 and 10, commonly in lesions involving the femur, tibia, humerus, and forearm [4]. Fracture rate in these locations decreased in adolescence, with even further decreases seen into adulthood [4]. Knowledge of this natural history makes it clear that if orthopedic procedures to stabilize long bones are to have an effect on outcome, they must be introduced very early in childhood to have an impact on the peak fracture rate [8, 9]. Similarly, once patients have reached adulthood, the chance of fractures becomes much lower, so less aggressive management to prevent a fracture may be indicated.

Surgical treatment consists of intralesional curettage and bone grafting with or without internal fixation [10–13]. Published reports, however, have documented recurrences after surgical curettage with or without bone grafting [14–18]. In addition, autografts have been found to subsequently turn into dysplastic tissue after getting incorporated into the host bone [8, 10]. Some authors therefore think that the best approach to FD management is a nonsurgical one [3].

It is evident from the literature that controversy exists regarding the approach to FD treatment. This paper is a retrospective cohort review reporting functional and radiological outcomes resulting from three minimally invasive surgical strategies employed by the authors for monostotic fibrous dysplasia (MFD), namely (1) plain open biopsy; (2) plain alpha-tricalcium phosphate (ATP) reconstruction using alpha-tricalcium phosphate filling paste (*Biopex*, Taisho Pharmaceutical Co., Ltd., Tokyo, Japan); and (3) prophylactic bridge plating.

## Patients and methods

From our institutional database, we identified 52 histologically verified cases of FD from 1989 to 2015. Prior institutional review board approval was obtained. Of the 52 patients, 29 patients seen before 2010 who were treated before we have started performing our minimally invasive surgical strategies, 4 patients with polyostotic disease, and 7 patients who presented with a bone deformity or pathologic fracture secondary to MFD as documented on radiographs were excluded, which left 12 patients for analysis. Surgical strategies for the 12 patients with MFD consisted of plain open biopsy in 5 patients, plain ATP reconstruction in 4 patients, and prophylactic bridge plating in 3 patients. There were nine (75%) men and three (25%) women (Table 1), with a median age of 38 years (range 11 to 65). Five (41.7%) lesions were in the proximal femur, two (16.7%) lesions each involved the femoral and tibial diaphyses, and one (8.3%) lesion each involved the distal humerus, radial midshaft, and proximal tibia. Mean follow-up period from the time of diagnosis was 88 weeks (range 7 to 205). Table 1 details patient age during surgery, sex, site of lesion, surgical strategy, and follow-up period for each of the 12 cases.

**Table 1:** Patient characteristics and outcomes

Case	Age	Sex	Site of lesion	Strategy	VAS score	Recovery time (days)	Radiological outcome	Postoperative complications	MSTS score
1	65	M	Proximal femur	Open biopsy	0	1	Partial resolution	Nil	30
2	48	F	Proximal tibia	Open biopsy	0	3	Partial resolution	Nil	30
3	28	M	Tibial diaphysis	Open biopsy	0	1	No change	Nil	30
4	61	M	Proximal femur	Open biopsy	0	1	Partial resolution	Nil	30
5	38	M	Distal humerus	Open biopsy	0	1	Partial resolution	Nil	30
6	38	M	Femoral diaphysis	ATP reconstruction	0	11	No change	Nil	30
7	39	F	Radial diaphysis	ATP reconstruction	0	92	No change	Nil	30
8	26	F	Proximal femur	ATP reconstruction	0	35	No change	Nil	30
9	38	M	Proximal femur	ATP reconstruction	0	192	No change	Nil	30
10	11	M	Tibial diaphysis	Bridge plating	0	143	Partial resolution	Nil	30
11	26	M	Femoral diaphysis	Bridge plating	0	3	Partial resolution	Nil	30
12	25	M	Proximal femur	Bridge plating	3	59	Progression	Nil	25

*M* male, *F* female, *ATP* alpha-tricalcium phosphate, *VAS* visual analog scale, *MSTS* Musculoskeletal Tumor Society

**Fig. 1:**
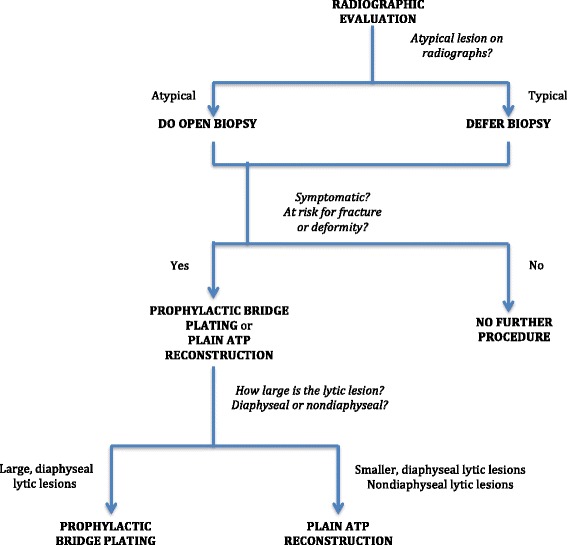
Surgical management algorithm for monostotic fibrous dysplasia

**Fig. 2:**
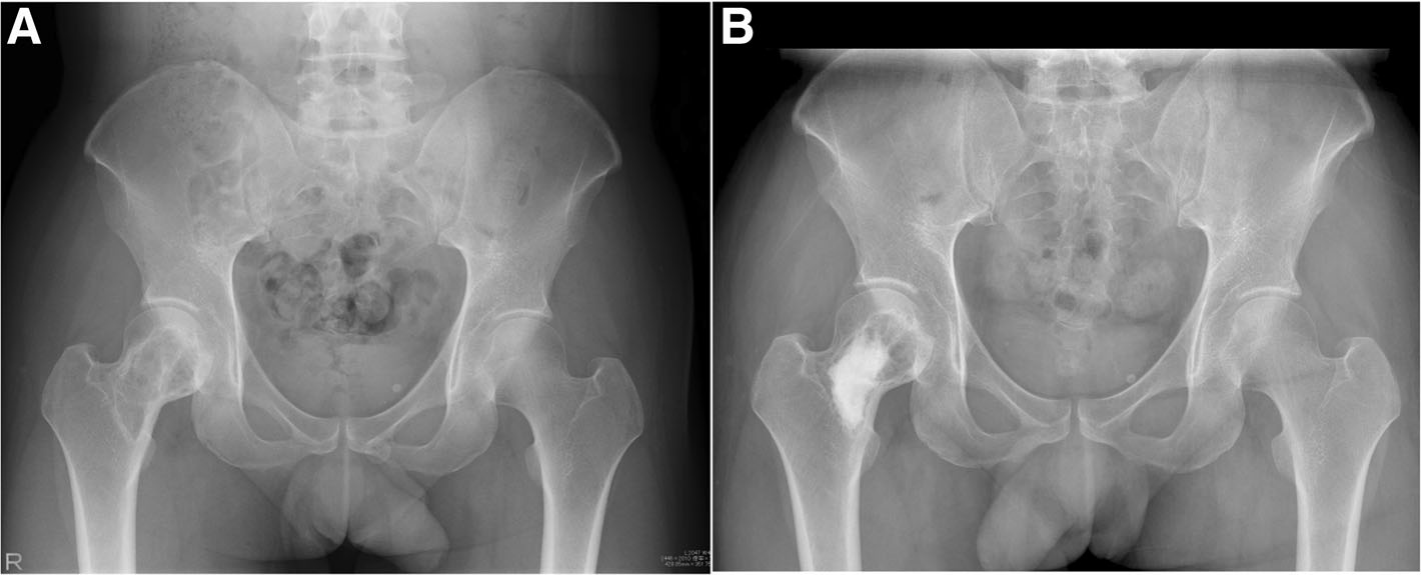
Patient 9: **a** AP radiograph showing proximal femoral lesion. **b** Postoperative AP radiograph at 48 weeks after plain ATP reconstruction showing no radiographic change of the lesion. Note the ATP reconstruction of only the lytic lesion, sparing nonosteolytic areas

**Fig. 3:**
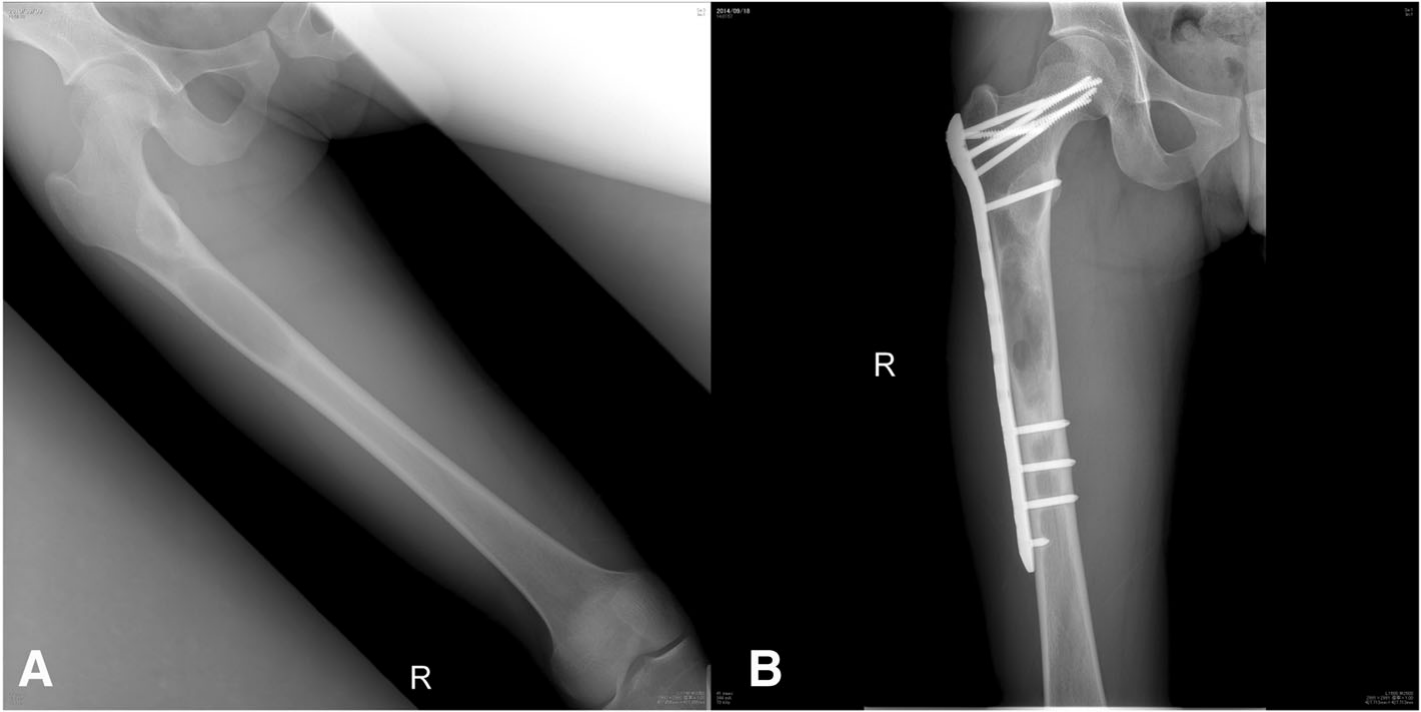
Patient 11: **a** AP radiograph showing femoral diaphyseal lesion. **b** Postoperative AP radiograph at 205 weeks after prophylactic bridge plating, showing partial resolution of the lesion

Since 2010, we had been following a surgical management algorithm for cases of MFD presenting with no deformity or pathologic fracture utilizing our minimally invasive surgical strategies (Fig. 1). For atypical lesions on radiographs that do not show findings of cortical thinning and expansion with endosteal scalloping, sclerotic margin, and a “ground glass” or cystic appearance [1], an open biopsy is performed. Asymptomatic lesions will have no further surgery after the open biopsy. For symptomatic cases, we perform plain ATP reconstruction on smaller diaphyseal and nondiaphyseal lytic lesions and prophylactic bridge plating on larger, diaphyseal lytic lesions, following establishment of the histopathological diagnosis. For the plain ATP reconstruction group, the procedure consisted of creating a cortical window over just the osteolytic area of the lesion and reconstructing the lytic bony defect with ATP (Fig. 2). For the prophylactic bridge-plating group, the surgery consisted of creating proximal and distal soft tissue windows and bridging the lesion with a submuscularly located plate inserted under fluoroscopic guidance, followed by insertion of proximal and distal locking screws without exposing the lesion (Fig. 3).

**Fig. 4:**
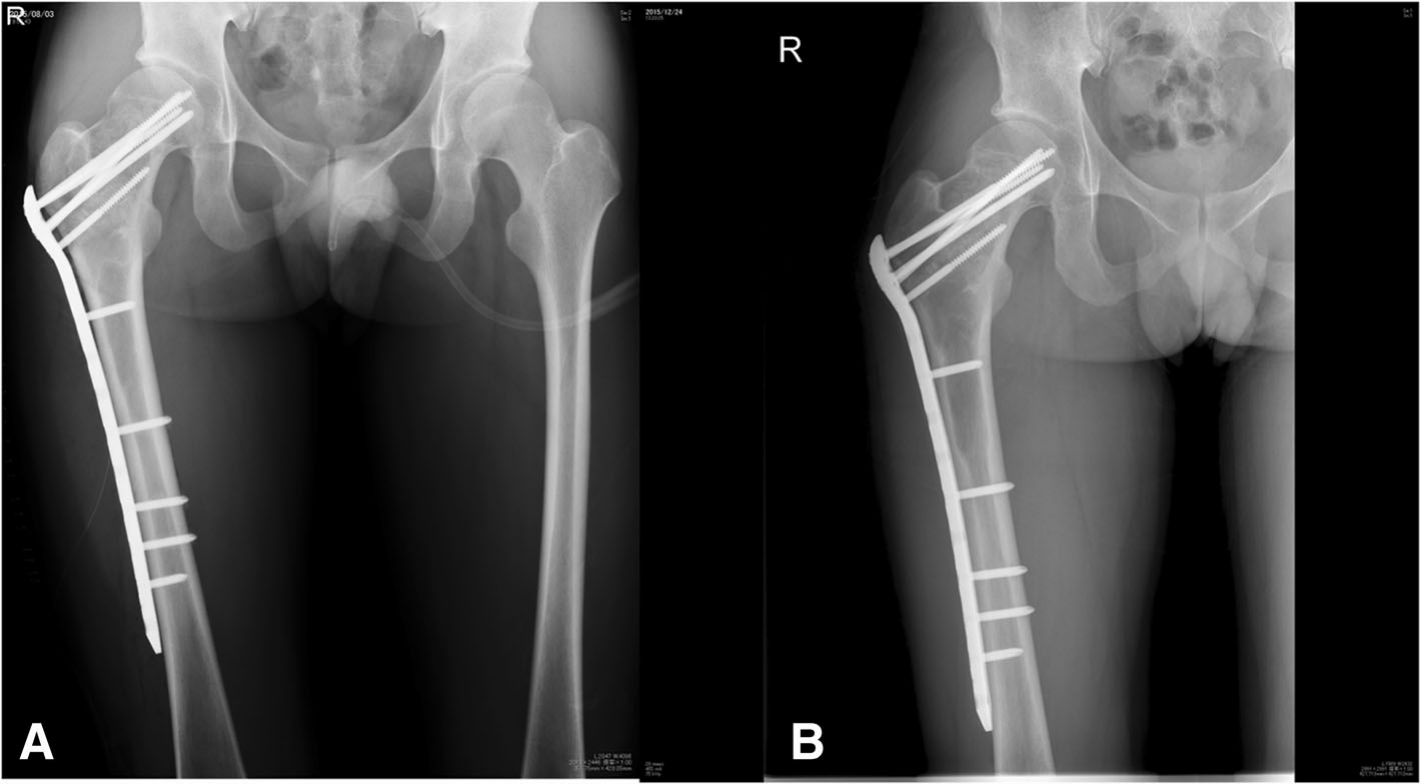
Patient 12: **a** AP radiograph immediately after prophylactic plating of proximal femoral lesion with proximal femoral locking plate (note proximal screws inserted through the lesion). **b** Postoperative AP radiograph at 18 weeks showing progression of the lesion

The authors utilized the system developed by Gaski et al. [19] for the radiographic analyses, which entailed comparison of preoperative and postoperative lesion status at last follow-up. Lesions were evaluated by observation of radiolucency, cystic appearance, endosteal scalloping, and both cortical contour and thickness. Postoperative lesion status was classified into one of four possible outcomes: *progression of lesion*, *no change*, *partial resolution*, or *complete resolution. Progression of lesion* was defined by increased radiolucency and cystic appearance, irregular cortical contour, and decreased cortical thickness with or without the development of endosteal scalloping (Fig. 4). *Partial resolution* was characterized by decreased radiolucency and cystic appearance, thickened cortices, lessened irregular cortical contour, and attenuation in the degree of endosteal scalloping if present preoperatively. Lesions were deemed *completely resolved* if there were no discernible radiographic abnormalities including the absence of lytic or cystic areas, with normal cortical borders and thickness.

Functional outcome criteria included pain status on last follow-up, time taken until return of full weight bearing (for the lower extremity lesion) or activity (for the upper extremity lesion), and occurrence of any complication postoperatively. Pain was measured using the visual analog scale (VAS). Recovery time was measured as the duration between the date of surgery and the date when full activity or weight bearing was resumed. The functional score was in accordance with the Musculoskeletal Tumor Society (MSTS) scoring criteria [20].

## Results

Results for all 12 cases are listed in Table 1. Four of the five plain open biopsy cases (80%) showed partial resolution on follow-up radiographs, while one case showed no radiographic change. All four cases of plain ATP reconstruction (100%) showed no radiographic change. Two of the three prophylactic bridge-plating cases (67%) showed partial resolution on follow-up radiographs, while one (33%) showed progression of the lesion.

Analysis of radiological outcomes per lesion site shows that plain open biopsy resulted in the partial resolution of lesions located in the proximal femur, while plain ATP reconstruction and prophylactic bridge plating resulted in no change and progression in this lesion site, respectively. For lesions in the femoral diaphysis, prophylactic bridge plating resulted in partial resolution, while plain ATP reconstruction resulted in no change. In the tibial diaphysis, prophylactic bridge plating resulted in partial resolution, while plain open biopsy resulted in no change. For the lesions involving the distal humerus and the proximal tibia, plain open biopsy resulted in partial resolution, while for the radial diaphyseal lesion, plain ATP reconstruction resulted in no change.

All patients had no pain at final follow-up, except for one case of prophylactic bridge plating for a proximal femoral lesion site (patient 12). None of the 12 cases had postoperative complications. Median recovery time was 1 day (range 1 to 3) for the plain open biopsy group, while the prophylactic bridge-plating and plain ATP reconstruction groups had longer median recovery times (59 days, range 3 to 143, and 52 days, range 11 to 192, respectively). MSTS functional scores at last follow-up were excellent for all 12 cases (mean 29.6, range 25 to 30).

## Discussion

The excellent functional scores and acceptable radiological outcomes of minimally invasive procedures (i.e., plain open biopsy, plain ATP reconstruction, and prophylactic bridge plating) found in this study must be remarkable when reminded of previous findings [14–18] showing unsatisfactory outcomes with curettage-incorporating surgical techniques in FD treatment. The concurrence of many published reports in showing less acceptable results with curettage-incorporating surgical approaches and the findings of this present study now challenge the present accepted principle of surgical care in treating FD lesions, that is, intralesional curettage and bone grafting with or without internal fixation [10–13].

Our study is not without limitations. First, being a retrospective study with a small number of cases, selection bias may be unavoidable. Therefore, findings of the present study may be considered preliminary, and further analyses with larger cohorts must be necessary. Second, our analyses compared the outcomes from our surgical strategies on different lesion sites. We recognize literature evidence that the proximal femur, which constitutes the majority of the skeletal sites affected by the lesion in our study, is hypothesized to become more susceptible to fatigue fracture when involved in FD, dynamically undergoing architectural alterations representing repeated reactions to fatigue stresses [21]. Third, the ages of the patients we included in our study were mostly beyond the time of physeal closure. Normalization of FD has been suggested to be a function of age, whereby as a lesion ages, mutant cells from the FD lesion fail to self-renew and their population gets depleted by apoptosis [22]. On the other hand, residual normal stem cells survive, self-renew, and enable the formation of normal bone [22]. And fourth, the varied follow-up periods preclude observation of possible long-term complications for the cases that had shorter follow-up.

Recognizing problems of possible recurrence and revisions with intralesional curettage, Demiralp et al. similarly propose prophylactic closed inflated intramedullary nailing without reaming as an alternative surgical option for MFD after having found improved pain scores and no postoperative progressions or complications in their series [23]. They used the *inflatable nail* (Fixion Intramedullary Nailing Systems, Disc-O-Tech Medical Technologies, Herzeliya, Israel) that has a short conical endpoint connecting four longitudinal bars, with a folded cylindrical chamber inside these bars and a single-direction valve that allows inflation of the chamber with fluid. After insertion of the nail, a special pump was connected to the valve and the nail was inflated with sterile saline with controlled pressure so that the nail adapts to the contour of the endosteal cortex. However, there could be limitations to using their nailing technique in patients with open femoral physes, who basically are at most risk for a pathologic fracture, for their series only included patients in the age range of 19 to 34 years. If prophylactic internal fixation to stabilize long bones is to have an effect on outcome, it must be introduced very early in childhood in order to have an impact on the peak fracture rate [8, 9].

The authors of this paper believe that allowing the patient to retain ambulation without walking aids, or to be able to use his or her affected arm or forearm as demanded by usual daily activities or work, should be a primary goal of MFD surgery for symptomatic cases. Intramedullary nails can be the most efficient devices for protecting the long bones from a pathologic fracture. Reamed intramedullary nailing has already been reported as an adjunctive stabilizing procedure after intralesional curettage for monostotic diaphyseal lesions in the lower extremities, showing improved clinical outcomes postoperatively [24]. But published reports having shown high recurrence and revision rates with intralesional curettage [14–18] make the results of Zhang et al.’s study [24], and consideration of this surgical treatment strategy, controversial.

The authors of this study present their results with the prophylactic, minimally invasive bridge plating for symptomatic, diaphyseal MFD. It is a reasonable surgical option for it provides optimal stabilization, does not violate tumor structure inside the bone, and may not require hardware removal upon disease resolution. The excellent MSTS and VAS scores with partial resolution of the lesions in the femoral and tibial diaphyses show promise for the technique as an alternative option for lesions similarly located in the lower extremity. To the authors’ knowledge, no previous study has focused on prophylactic bridge plating of lower extremity long bone diaphyses affected with MFD in symptomatic patients. In contrast to Demiralp et al.’s prophylactic closed inflated unreamed intramedullary nailing procedure [23], the prophylactic bridge-plating technique does not violate the physes, thereby serving as a suitable option to stabilize symptomatic monostotic lower extremity diaphyseal lesions in the pediatric age group. Our youngest patient (11 years) with a large MFD of the tibial diaphysis, however, had a long recovery time (5 months) following prophylactic bridge plating. Despite a longer rehabilitation time with large lesions compared to the use of a load-sharing device like the intramedullary nail especially in younger patients with active disease, a long-lasting stabilization without the need for implant removal, in addition to its physeal-sparing advantage, makes prophylactic bridge plating an acceptable alternative procedure for the described natural course of MFD [8, 9, 25].

In the present study, one prophylactic plating case of a proximal femoral MFD (that of patient 12) was seen on follow-up radiographs to have a progressing lesion. Compared to the other two, this case apparently had proximal screws inserted through the lesion, thereby not being surgically treated, in the strictest sense, with “bridge” plating. Lindner et al., in a review of 70 cases of FD with a median follow-up of 6.5 years and managed with either intralesional curettage or marginal en bloc excision, have found a higher revision rate for proximal femoral lesion sites [16]. It was suggested that the proximal femur becomes more susceptible to fatigue fracture when involved in FD, dynamically undergoing histological changes representing repeated reactions to fatigue stresses [21]. The repeated morphological changes that weaken this skeletal segment may suggest inherently poor surgical outcomes for a proximal femoral lesion site, for which a nonoperative treatment strategy, until FD normalization is reached, might be the best option to avoid the surgical complications. Our results show partial resolution of all two proximal femoral lesions managed with plain open biopsy (patients 1 and 4). Internal fixation following either intralesional curettage and allograft impaction grafting [26] or minimal curettage and autogenous fibular cortical strut grafting [27] has also been shown to be effective treatment for proximally located femoral FD lesions in two case series.

Monostotic lesions remain active only until skeletal maturity. The authors hereby propose a noninvasive surgical strategy for asymptomatic proximal femoral lesions, that is, just plain open biopsy for the sole purpose of reaching a definitive diagnosis. Plain open biopsy, as shown, allows patients to return to their usual activities as early as 1 day after the procedure. Other reported strategies [26, 27] mentioned in the previous paragraph seem controversial for the sole reason that the techniques utilize intralesional curettage. A not-too-invasive procedure that gives the fastest recovery time must be a suitable treatment for asymptomatic MFD of the proximal femur.

For symptomatic MFD of the proximal femur, the authors’ strategy of doing just plain ATP reconstruction may work to control the disease until normalization is reached. All lesions managed with plain ATP reconstruction had no radiologic change at last follow-up, which could mean that although the lesions do not appear to resolve following plain ATP reconstruction, the latter may work to promote disease control throughout the active phase until FD normalization is reached. Both the two proximal femoral FD cases treated with plain ATP reconstruction had excellent functional scores with no postoperative complications or pain. However, one case had an unsatisfactorily long recovery time (7 months), reflected by the long rehabilitation period following plain ATP reconstruction of a large lytic defect. One prophylactic strategy can be that by Gaski et al., utilizing retrograde unreamed flexible nailing with titanium elastic nails [19]. Although the procedure was used for a pathologic fracture case through a proximal femoral lesion, long-term assessment showed complete fracture healing with full range of motion in the affected hip and no postoperative complications [19]. Similar to prophylactic bridge plating, this strategy can be opted for the younger patients with open femoral physes to provide stable support throughout the active phase of the disease. Its unreamed technique and avoidance of intralesional curettage makes retrograde flexible nailing a suitable minimally invasive option for symptomatic monostotic lesions in areas not amenable to “bridge” plating, like those located in the proximal femur.

## Conclusion

Our minimally invasive surgical strategies have been shown to limit radiological progression in 11 (92%) of the 12 cases analyzed. This could be the reflection of a generally favorable prognosis for older-aged patients who constituted the majority in our study. However, we believe that our minimally invasive procedures, by preserving normal bone to maintain stability, may work to promote disease normalization through time while keeping patients functional. Therefore, doing plain open biopsies for asymptomatic disease; prophylactic bridge plating for symptomatic, large diaphyseal lesions; and plain ATP reconstructions for both small and large nondiaphyseal symptomatic lesions may be acceptable alternative options to curettage-incorporating procedures for MFD.

## Abbreviations

AP: Anteroposterior
ATP: Alpha-tricalcium phosphate
FD: Fibrous dysplasia
MFD: Monostotic fibrous dysplasia
MSTS: Musculoskeletal Tumor Society
VAS: Visual analog scale
